# Pathogenesis and precision medicine for predicting response in inflammatory bowel disease: advances and future directions

**DOI:** 10.1136/egastro-2023-100006

**Published:** 2024-01-18

**Authors:** Robert D Little, Thisun Jayawardana, Sabrina Koentgen, Fan Zhang, Susan J Connor, Alex Boussioutas, Mark G Ward, Peter R Gibson, Miles P Sparrow, Georgina L Hold

**Affiliations:** 1 Department of Gastroenterology, Alfred Health, Melbourne, Victoria, Australia; 2 Faculty of Medicine, Nursing and Health Sciences, Monash University, Clayton, Victoria, Australia; 3 Microbiome Research Centre, University of New South Wales, Sydney, New South Wales, Australia; 4 South West Sydney Clinical Campuses, Medicine & Health, University of New South Wales, Sydney, New South Wales, Australia; 5 Department of Gastroenterology, Liverpool Hospital, Sydney, New South Wales, Australia

**Keywords:** Ulcerative Colitis, Inflammatory Bowel Diseases, Crohn's Disease, Gastrointestinal Microbiome

## Abstract

The pathogenesis of inflammatory bowel disease (IBD) is complex and multifactorial. Undertreated disease has substantial individual and societal consequences. Current patient classification and subsequent positioning of IBD therapy are based on crude, readily accessible clinical data. These broad parameters are unlikely to reflect underlying molecular profiles and may account for the observed heterogeneity in treatment response. Precision medicine offers identification and integration of molecular profiles into clinical decision-making. Despite several promising scientific and technological advances, the pathogenesis and targetable molecular drivers of IBD remain incompletely understood. Precision medicine therefore remains aspirational. This comprehensive narrative review describes our current understanding of IBD pathophysiology, highlights preliminary genetic, immunological and microbial predictors of treatment response and outlines the role of ‘big data’ and machine learning in the path towards precision medicine.

## Introduction

 Inflammatory bowel disease (IBD) comprises a group of chronic, relapsing, immune-mediated disorders including both ulcerative colitis (UC) and Crohn’s disease (CD).^1 2^ The prevalence of IBD is increasing worldwide.^3^ Analyses from the Global Burden of Disease Study across 195 countries over a 27-year period reported an estimated 6.8 million cases of IBD globally with an increase in age-standardised prevalence from 79.5 (75.9–83.5) per 100 000 in 1990 to 84.3 (79.2–89.9) per 100 000 people in 2017.^3^ The health economic consequences of this are substantial.^4^,^6^ In Europe, the mean annual healthcare costs for prevalent CD and UC were US$12 439 and US$7224, respectively. In North America, these values increased to mean annual healthcare costs of $17 495 for CD and $13 559 for UC. The primary driver of these annual costs appeared to be related to greater access to advanced medical therapies, highlighting the future benefit of rational, tailored drug selection for each patient.^6^

The geographical distribution in both incidence and prevalence is not equal. A systematic review of 147 population-based studies reported a prevalence of approximately 0.3% across North America, Australia, New Zealand and many Western European countries.^7^ While the prevalence is high, the incidence is stabilising in Western countries.^7^ Developing nations across Asia, Latin America and Africa appear to be facing an acceleration in IBD incidence, correlating with increased industrialisation and Westernisation.^8^,^10^

Gut microbial structure and function are influenced by dietary intake and may account for the association between diet and IBD.^11 12^ Rural and remote communities have greater gut microbial diversity and richness than individuals from developed nations.^13^,^15^ In contrast to the Western diet, higher fibre and raw plant intake in these communities may explain some of these observations. In population-based studies, the onset of UC has been associated with higher animal protein, trans and omega-6 fatty acid and, perhaps, sweetened beverage intake.^16^,^23^ In CD, elevated protein intake and ultra-processed foods are associated with disease development^20 24 25^ and a higher intake of fibre, dairy products, docosahexaenoic acid and certain polyphenols appear to be protective.^1826^,^30^ Further characteristics of a Westernisation ‘exposome’ are also associated with alterations in gut microbiota and IBD onset such as antibiotic exposure,^31^,^34^ smoking,^35^ air pollutants^36 37^ and excessive hygiene.^38^,^41^ However, the pathogenesis of UC and CD is complex and incompletely understood. Both the development and course of the disease appear to involve interconnected, overlapping contributions from a genetic predisposition, impaired intestinal barrier function, an aberrant host immune response, altered gut microbiota composition and function, and the environmental factors discussed above.^42 43^

**Figure 1:**
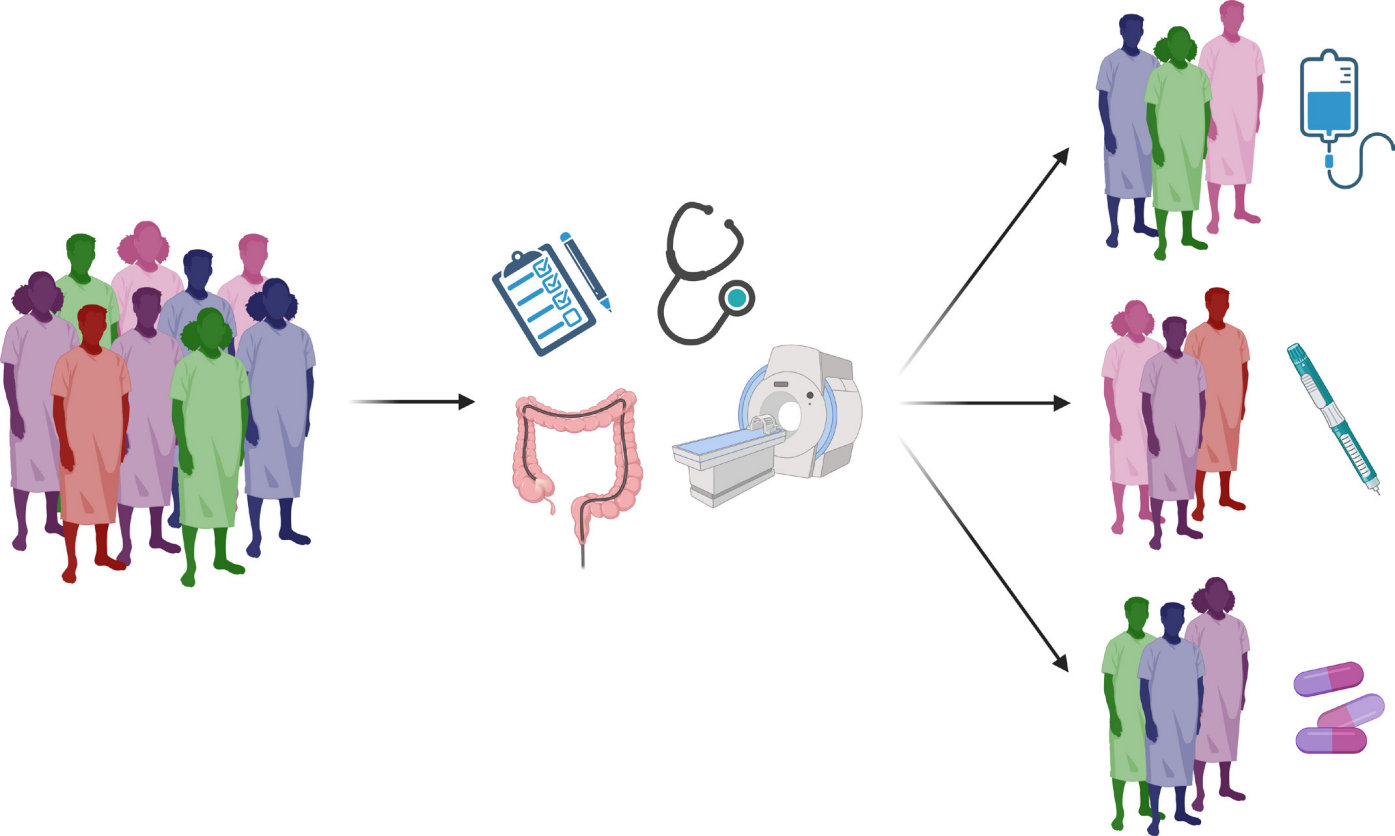
An illustration of the current imprecise approach to selecting IBD therapy. In this approach, patients are assessed using crude clinical, endoscopic and radiological evaluation. Subsequent categorisation results in inaccurate and heterogeneous patient phenotyping and thereby imprecise selection of IBD therapy. Created with BioRender.com. IBD, inflammatory bowel disease.

IBD is incurable and often requires long-term immunosuppressive therapy to reduce the rate of progression and occurrence of complications.^44^,^47^ Despite a growing therapeutic armamentarium, there remains a ‘ceiling effect’ in rates of response and remission in patients with IBD.^48^ In the major registration trials, only 15–50% of patients responded to induction of available biological and small-molecule therapies.^49^,^62^ Furthermore, in regard to drug selection, there is a paucity of available head-to-head trials in IBD to inform management decisions.^63^ Evidence guiding the selection of biological or small-molecule agents is therefore restricted to network meta-analyses, observational studies and expert opinion.^64^,^66^ In clinical practice, choosing between agents is often informed by broad clinical, biochemical, radiological and endoscopic phenotyping of patients^67^,^69^ (figure 1).

**Figure 2:**
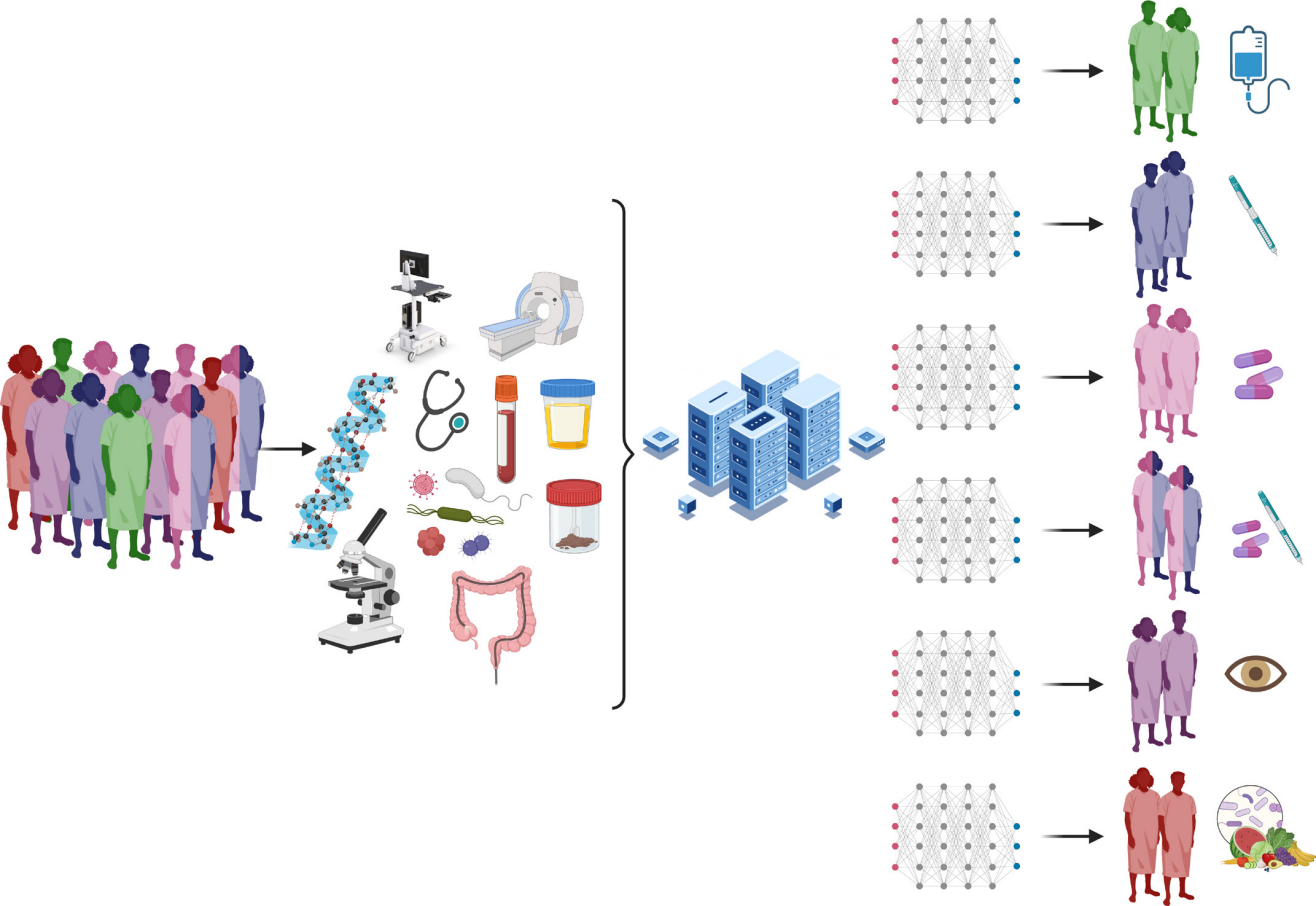
An illustration of the future of precision medicine and informed selection of IBD therapy. In this approach, patients are assessed using a combination of clinical and molecular profiling, incorporating genetic, immunological and microbial evaluation. Complex raw data are interpreted by omics-based network medicine, allowing accurate molecular profiling of patient groups and informed selection of a therapeutic agent, combination therapy, observation or novel dietary or microbial interventions. Created with BioRender.com. IBD, inflammatory bowel disease.

Scientific advancements including genome-wide association studies (GWAS), whole-genome sequencing, shotgun metagenomics and additional high-throughput omics analysis including transcriptomics, proteomics and metabolomics as well as sophisticated bioinformatics have provided greater insight into IBD pathophysiology.^4870^,^72^ However, integration of these findings to better inform selection of therapeutics remains in its infancy. A greater understanding of the underlying disease pathogenesis may eventually inform more sophisticated, personalised management strategies in the pursuit of ‘precision medicine’.^73^ For the purpose of this review, we interpret precision medicine as an approach that ‘seeks to improve stratification and timing of healthcare by using biological information and biomarkers on the level of molecular disease pathways, genetics, proteomics as well as metabolomics*’*.^74^ Precision medicine in oncology is established with genomic profiling in particular guiding treatment of many tumour types.^75 76^ For example, in the treatment of non-small cell lung cancer, broad platinum-based chemotherapy regimens may be avoided by targeting readily identifiable driver mutations such as ALK, BRAF, EGFR and ROS1.^75^ Similar biomarkers informing selection of targeted therapeutics exist for breast cancer (HER2 expression, trastuzumab), chronic myeloid leukaemia (BCR–ABL1 fusion, imatinib), metastatic melanoma (BRAF V600E, BRAF and MEK inhibitors), chronic lymphocytic leukaemia (17p deletion, venetoclax) and gastrointestinal stromal tumours (KIT expression, imatinib).^77^ Despite recent interest from the European Crohn’s and Colitis Organisation’s Scientific Steering Committee,^73 78^ there are currently no molecular correlates that determine the management of non-monogenic IBD. Pursuing precision medicine requires more accurate molecular profiling of the underlying environmental, genomic, epigenomic, microbial, metabolomic and immunological drivers of IBD in each individual^78^ (figure 2). This ‘multiomics’ network medicine approach then relies on machine-based biostatistical analysis to interpret the volume and complexity of data.^71 79^

This comprehensive narrative review will summarise our current understanding of the genetic, immunological and microbial drivers of IBD. After reviewing each pathogenetic category, this review will highlight novel predictors of treatment response. Contemporary evaluation of molecular biomarkers in IBD requires an understanding of the principles and importance of incorporating machine-based bioinformatics and collaborative research. These will be discussed alongside the future directions for precision medicine in IBD required to inform optimal, personalised management strategies to improve the quality of life of patients with IBD.

### Genetics

IBD has long been associated with a heritable risk, particularly for CD.^80 81^ GWAS have identified approximately 240 IBD risk variants to date. These include polymorphisms in genes encoding regulatory receptors at the intestinal epithelial barrier (eg, nucleotide-binding oligomerisation domain 2 (NOD2)); genes encoding proinflammatory cytokines or their receptors (eg, tumour necrosis factor (TNF) superfamily member 15, TNF-α, interleukin (IL)-23 receptor (IL-23R)); and genes encoding regulatory, anti-inflammatory cytokine receptors (eg, IL-10 receptor subunit α) or cell death pathway proteins (eg, X linked inhibitor of apoptosis).^82 83^ The relative frequency and contribution of each risk locus vary according to patient ethnicity.^84^ However, outside of monogenic IBD, only an estimated 13.6% of disease variance in CD and 7.5% in UC can be explained by inheritance of known risk loci.^85^ Furthermore, monozygotic twin studies demonstrate only modest concordance in the development of CD (20–55%) and UC (6.3–17%) which drops to as low as 3.6% and 6.3% for dizygotic twins, respectively.^86^ Reanalysis of existing biobanks using newer technologies such as next-generation sequencing (NGS) may allow further genetic insight into IBD pathogenesis.^87^ NGS enables faster, deeper genomic evaluation and may identify rare, low-frequency variants not detected by standard GWAS.^88^

While markers such as thiopurine methyltransferase and Nudix hydrolase-15 polymorphisms^89^,^93^ are integrated into clinical practice for prediction of drug toxicity,^89^,^93^ incorporation of germline mutations to predict direct drug efficacy has not yet been validated or routinely adopted.^94^ The majority of existing genetic analyses in IBD have been performed to predict response to anti-TNF therapies, with less data on small molecule and alternative biological classes. Preliminary work suggests that homozygosity for high-risk IL-23R variants^95^ and polymorphisms at the Fas ligand locus^96^ and IBD5 locus^97^ have been associated with anti-TNF response. In contrast, the more established NOD2,^98 99^ TNFR1 and TNFR2^100 101^ polymorphisms appear to have no association with response to anti-TNF therapy. To date, perhaps the most promising genetic insight into treatment response relates to the prediction of antidrug antibody formation, a common cause for secondary loss of response to anti-TNF therapy.^102^,^106^ Application of whole-exome sequencing of DNA extracted from pretreatment blood samples from a large cohort of patients commencing infliximab or adalimumab has identified an association between the HLA-DQA1*05 haplotype and a near doubling of the risk of developing immunogenicity to anti-TNF agents.^104 107^ A meta-analysis, published in abstract form only, supports the association between HLA-DQA1*05 carriage and immunogenicity to anti-TNF agents.^108^ Despite the high prevalence of HLA-DQA1*05 in Europe and North America,^108 109^ similar to many existing genetic predictors of response, prospective external validation is limited and uptake in routine clinical practice is variable.

### Intestinal barrier and mucosal immunity

The intestinal epithelium is a complex, dynamic barrier comprising a single layer of cells connected by tight junctions.^110 111^ The majority of intestinal epithelial cells are columnar epithelial cells (enterocytes) responsible for nutrient absorption.^112^ Secretory intestinal epithelial cells include goblet cells, Paneth cells and enteroendocrine cells.^112^ Goblet cells secrete a protective mucous layer containing antimicrobial peptides produced by Paneth cells as well as secretory IgA produced by plasma cells within the lamina propria.^113 114^ Controlled transcytosis of luminal microorganisms mediated by microfold cells, dendritic cells and macrophages regulates innate and adaptive mucosal immunity. Subepithelial stromal cells, including fibroblasts and myofibroblasts, reside within the lamina propria and play important roles in wound healing, fibrosis and a complementary role in mucosal immunity.^115^ A reduced mucin layer or disruption of the epithelial barrier may increase intestinal permeability and drive inflammation via uncontrolled passage and handling of microbial antigens in both CD and UC.^116^,^120^ Indeed, data from the Crohn’s and Colitis Canada Genetic Environmental Microbial (CCC GEM) Project demonstrate that increased intestinal permeability, as measured by urinary fractional excretion of lactulose and mannitol, has been observed in previously healthy relatives of patients with CD prior to eventual development of CD^121^—highlighting the potential early role of the intestinal barrier in the pathogenesis of CD.

Immune cells within the gastrointestinal system are primarily located in secondary lymphoid structures such as Peyer’s patches, interspersed between columnar intestinal epithelial cells, residing within mesenteric lymph nodes or embedded in underlying connective tissue.^42^ In healthy intestinal mucosa, the mucosal immune compartment supports homeostasis via maintenance of anti-inflammatory pathways. Downregulation of the immune response occurs via mediators such as IL-10, transforming growth factor β (TGF-β), retinoic acid and expansion of forkhead box P3 (FOXP3+) regulatory T (Treg) cells.^122 123^ Aberration of any of these complex, interconnected innate and adaptive signalling pathways may contribute to the pathogenesis of IBD.

In IBD, increased intestinal permeability increases antigen and adjuvant exposure.^119 124^ Activated mucosal proinflammatory macrophages engulf invading microbiota and secrete a range of proinflammatory cytokines including TNF, IL-6, IL-1β, IL-23, IL-12 and chemokine ligand 2.^125^ Antigen presentation to CD4+ T cells leads to predominant differentiation and expansion of T helper (Th) 1 and Th17 cells.^126 127^ Alongside group 1 and group 3 innate lymphoid cells (ILCs), Th1 and Th17 propagate an inflammatory feedback loop via secretion of chemokines and net proinflammatory IL-17A, IL-17F, IL-22 and interferon-γ.^127 128^ Active IBD is also associated with a relative increase in IgG in contrast to the protective IgA predominance of healthy intestinal mucosa.^129 130^ This anti-commensal IgG appears to drive increased IL-1β production and a shift to type 17 immunity in colonic mucosa of patients with UC.^131^ CD4+ and CD8+ tissue-resident memory T cells within the intestinal epithelium and lamina propria are also activated and further propagate the innate and adaptive immune response.^132^,^134^ Furthermore, anti-inflammatory compensatory mechanisms are reduced with lower Treg cell activity and associated reductions in anti-inflammatory IL-10 and TGF-β.^135^

Interrogating functional epithelial and mucosal immune cell gene expression using transcriptomics has generated promising preliminary findings. RNA sequencing (RNA-seq) allows high-throughput analysis of the entire transcriptome within a particular sample.^136^ More recently, RNA-seq has been performed at the single-cell level (scRNA-seq). scRNA-seq allows identification and comparison of the transcriptomes of individual cells within a heterogeneous sample.^137^ With increasing access and affordability, scRNA-seq is being increasingly applied to the prediction of therapeutic response in IBD.^138^ For example, application of scRNA-seq and multiparameter mass cytometry techniques allowed identification of a unique, interconnected cellular group in inflamed ileal tissue associated with anti-TNF non-response in patients with CD.^130^ Termed the GIMATS module, this cellular profile consisted of IgG plasma cells, inflammatory mononuclear phagocytes, activated T cells and stromal cells.^130^ Transcriptomic analysis using an mRNA microarray platform has also been used to predict response to anti-TNF in patients with UC with a predicted sensitivity of up to 95%.^139^ Similarly, transcriptomic data from intestinal mucosal biopsy samples of patients with UC and CD identified that a transcriptional module co-expressed with a recently implicated cytokine, oncostatin M, predicted non-response to anti-TNF (area under the receiver operator curve (AUROC) 0.99).^140^ Further studies measuring mucosal gene expression have found a number of accurate transcriptional signatures associated with response to anti-TNF therapy,^139 141^ including triggering receptor expressed on myeloid cells 1 (TREM1; AUROC 0.77, p=0.003)^142^ and IL-13RA2 expression—found to be strongly associated with anti-TNF non-response (AUROC 0.9, p<0.001).^143 144^ On a larger scale, analysis of publicly available datasets from registration trials in IBD found that a higher abundance of plasma cells and macrophages was associated with anti-TNF non-response.^145^ These findings highlight the utility of a collaborative ‘big data’ approach to advancing precision medicine.^74^ Aside from TREM1,^142^ it is not yet clear whether immunophenotypical predictors of response are specific to anti-TNF therapy. For example, transcriptomic analysis of mucosal biopsies in 41 patients with UC found that almost two-thirds of the genes that predicted response to vedolizumab also predicted response to infliximab.^146^ Further research into predictors of other biological drug classes and small molecules is necessary to inform positioning of these agents for individual patients.

### Gut microbiota

There is an increasing acceptance of the influence of gut microbiota in the pathogenesis and disease course of IBD.^147 148^ The healthy human gut is colonised by an estimated 100 trillion bacterial, viral and fungal microorganisms with an increasing density moving distally from the stomach to the colon.^149^ Bacteria are the most abundant and the majority of these organisms belong to one of four dominant phyla: Bacteroidota (Bacteroidetes), Bacillota (Firmicutes), Pseudomonadota (Proteobacteria) and Actinomycetota (Actinobacteria).^150^,^152^ Diversity in gut microbial signatures between individuals is common and is likely secondary to a bidirectional relationship between environmental exposures such as diet and underlying host genetics.^153^ Germ-free and antibiotic-treated animal models provide crucial evidence for the role of gut microbiota in the development and maturation of host immunity.^154^ Germ-free mice have impaired development of gut-associated lymphoid tissue such as Peyer’s patches,^155 156^ reduced IgA production,^157 158^ reduced ILCs,^159 160^ altered Th cell expression^161 162^ and reduced colonic FOXP3+ Treg cells.^163^,^165^ A number of these immune aberrancies are also partially reversed by introduction of colonising microorganisms.^162163 165^,^167^

**Figure 3:**
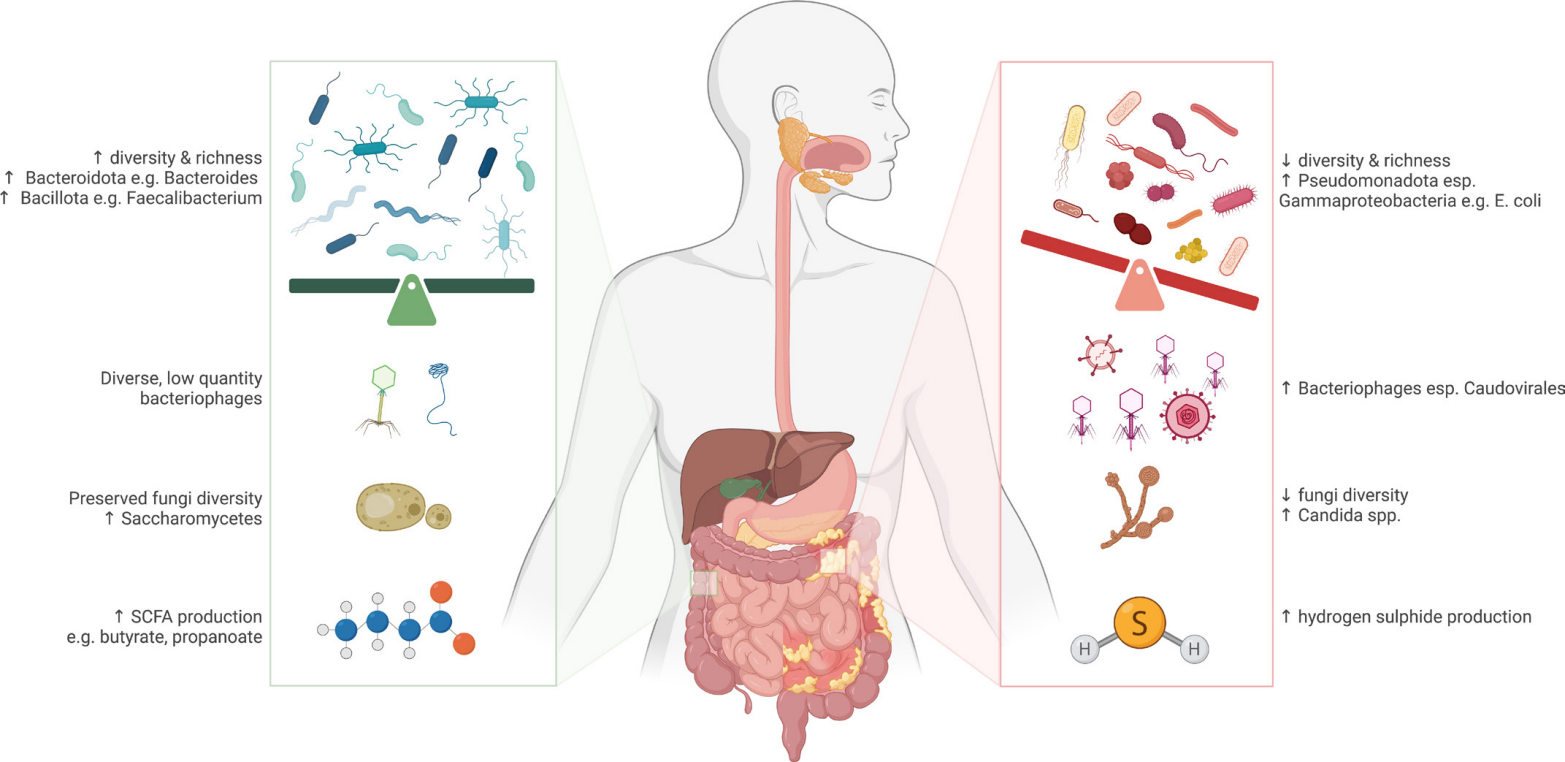
Major community and species-specific alterations in gut microbiota and metabolites associated with active inflammatory bowel disease (right) relative to healthy bowel (left). Created with BioRender.com. SCFA, short-chain fatty acid.

Dysbiosis describes disruption of a balanced microbial ecosystem.^168^ While dysbiosis is associated with IBD onset and disease activity, human data confirming a causal relationship are scarce. Broad compositional microbial changes in patients with IBD include reduced bacterial, fungal and viral diversity and richness.^169^ More specific microbial changes associated with IBD include depletion of healthy commensal bacterial groups such as Bacteroidota and Bacillota and expansion of proinflammatory classes within the Pseudomonadota phyla, such as Gammaproteobacteria (eg, *Escherichia coli*) as well as increased bacteriophage numbers (eg, Caudovirales) and pathogenic Ascomycota (eg, *Candida albicans*) (figure 3).^170^,^182^ Whether these findings are a cause or consequence of intestinal inflammation in humans is yet to be determined.^183^ However, a pathogenic role is suggested by animal studies demonstrating that direct or passive faecal transfer from mice or humans with colitis to healthy mice can induce susceptibility to intestinal inflammation.^135 184^ Disturbance of the nutritional, homeostatic and immunomodulatory functions of commensal microbiota provides mechanistic insights into a possible pathogenic association.^185^ Nutritional roles include production of water-soluble B vitamins,^186^ vitamin K^187 188^ and short-chain fatty acids (SCFAs) such as acetate, propionate and butyrate.^189^ At moderate levels, butyrate plays a positive role in preserving epithelial integrity, colonocyte growth and maintaining mucosal immunity.^189^,^192^ Patients with active IBD appear to have reduced levels of butyrate-producing bacteria^193^ and a higher proportion of sulfate-reducing bacteria, which may contribute to mucosal inflammation via excessive production of hydrogen sulfide.^194^,^196^ Additional data from the CCC GEM Project demonstrate increased faecal proteolytic and elastase activity in patients with UC prior to their IBD diagnosis, reflecting altered functional activity of microbiota compared with healthy matched controls.^197^ Disruption of these secondary metabolic activities may compromise intestinal barrier function, lead to aberrant mucosal immune responses and provide a plausible mechanism linking microbial dysbiosis to the development of IBD.^198^,^201^

For future clinical integration, molecular biomarkers for the prediction of treatment response should be readily accessible, non-invasive and inexpensive.^78^ A number of previous studies have investigated the utility of gut microbial ‘signatures’ predictive of treatment response in IBD either in easily accessible faecal samples or mucosal sampling. In general, higher microbial diversity, fewer mucus-colonising bacteria, higher abundance of SCFA-producing bacteria and lower abundance of ‘proinflammatory’ bacteria are associated with favourable response to anti-TNF agents,^202^,^207^ vedolizumab^208^ and ustekinumab.^209^ More granular data at a species level may outperform broader taxonomic profiles at the level of genus or class.^208^ Predictive software models, informed by such high-quality data, have reasonable accuracy in predicting therapeutic response for both vedolizumab and anti-TNF therapy.^208^ Evaluating microbial metabolite production using metabolomic techniques may provide additional predictive capacity and provide insight into the functional relevance of changes to community structure. For example, production of butyrate or substrates involved in butyrate synthesis has been associated with response to anti-TNF therapy.^203^ However, the proportion of observed alterations to microbial structure and function that is due to medication effect, intestinal inflammatory burden and dietary modification rather than specific to treatment response remains unknown.^210^ Large longitudinal observational cohorts of patients with IBD and healthy controls with serial biosampling, such as CCC GEM^121 197^ and the Australia IBD Microbiome Study,^211^ may allow greater insight into the pathogenic role and predictive capacity of perturbed gut microbiota in the course of IBD.

While using multiomics to inform precision medicine in IBD is an exciting prospect, few of the above observations have been validated in independent, prospective populations and none of the above genetic, immunological or microbial predictors of treatment response are incorporated into routine clinical practice. A collaborative ‘big data’ approach to precision medicine is likely necessary to advance towards precision medicine in IBD.

### Big data and machine learning

Interpreting the volume and complexity of the above biological data requires sophisticated biostatistical techniques. Traditional human-supervised statistical methods have been inadequate to meaningfully unlock the pathogenesis of IBD. Rather, interpretation of multilayered molecular data requires systems biology and machine learning.^71 74^ Systems biology refers to mathematical network modelling of complex biological systems and their response to perturbation.^212^ In handling the data-rich nature of biological systems, machine learning is an invaluable tool that can uncover novel insights from large datasets thus model the structure and dynamics of biological networks. Machine learning, a subset of artificial intelligence, refers to the development of computational algorithms that are able to learn from data to better detect patterns and adjust decisions without the need for explicit programming.^213^ Deep learning is a specialised type of machine learning that is capable of identifying highly complex patterns within and between large datasets using deep neural networks with multiple layers.^214^ Deep learning techniques allow greater flexibility and higher capacity with millions of trainable parameters. However, these models require training on large, carefully ‘curated’ datasets with low confounding.^214^ If supplied with accurate, high-volume data, these approaches will allow precise, sophisticated molecular categorisation of patients and may predict therapeutic response.

Standard machine learning techniques such as random forests, logistic regression and support vector models as well as more advanced deep learning models such as neural networks have been applied to genomics data from large IBD consortiums with both identification of new variants and confirmation of previously identified genetic variants associated with both CD and UC.^215^,^217^ Machine learning techniques applied to over 30 000 patients with IBD (17 379 CD, 13 458 UC) and 22 000 controls accessed via the International IBD Genetics Consortium generated high-performance predictive models for identification of CD and UC (AUROC 0.86 and 0.83, respectively).^215^ Similarly, genomic, transcriptomic, proteomic and microbiome data from smaller cross-sectional and longitudinal cohorts analysed by standard and advanced machine learning techniques have generated promising preliminary results.^207^,^209218^ For example, 41 genes associated with IBD were identified via application of sequential novel computational techniques on gene expression profiles of just 75 patients with IBD and 42 healthy controls accessed through a public genomics data repository (Gene Expression Omnibus).^222^

Deep learning models are already making progress in automated interpretation of endoscopic disease activity in UC.^223^,^225^ After training on a large dataset of >40 000 endoscopic images and 6885 biopsy results, model processing of endoscopic images alone could also predict histological remission in UC with an accuracy of 93%.^226^ The same deep neural network model could predict subsequent patient outcomes in UC based on endoscopic images alone.^227^ However, predicting treatment response is likely to be better informed by the combination of molecular and clinical data. VedoNet, a neural network algorithm informed by longitudinal clinical and microbiome data from just 85 patients with IBD, accurately predicts early clinical response to vedolizumab (AUROC 0.87) as well as anti-TNF response in a smaller validation cohort.^208^ As expected, the combination of clinical and molecular data performed better in predicting therapeutic response than either parameter alone.

Predictive modelling generated by machine learning has not yet entered routine clinical practice in the management of IBD. Greater utilisation and eventual incorporation of machine learning techniques require significant computing power and large, accessible datasets. Furthermore, as reported in translational cancer research, data may be incomplete, restricted by release policies and access costs or limited by inconsistencies in measurement generated by varying experimental platforms.^79 228^

### Future directions

Applying precision medicine to predict therapeutic response is likely to revolutionise patient care in IBD. However, there are several known barriers to achieving this goal. Coordinated reanalysis of existing, well-characterised datasets with newer experimental or analytical techniques is a cost-effective initial step. Longitudinal biobanking within both registration trials and regional healthcare systems would also overcome many of the limitations raised by underpowered existing cohorts.^75^ Improving the quantity and homogeneity of data drawn from available samples requires more uniform and inexpensive multiomic experimental techniques. These data must then be readily accessible via collaborative research agreements and data sharing platforms to provide the volume and completeness to allow training of and interpretation by sophisticated, unsupervised deep learning models.^77^ Furthermore, given the variance in environmental exposures across the globe as well as possibly distinct genetic risk profiles across ethnicities, efforts must be made to incorporate diverse populations in multiomics analyses to ensure generalisability of biomarker discovery. How best to incorporate the impact of the exposome, including early antibiotic exposure, diet, pollutants and smoking, on disease course and treatment response remains unclear.

Once identified, successful integration of novel predictive biomarkers into routine care of IBD management requires careful consideration. Varied uptake and application of precision medicine in oncology provide a cautionary lesson. Despite established efficacy, genetic testing for targetable mutations is often underused and varies across regions and socioeconomic backgrounds.^229^ Clinical guidelines, education and sophisticated decision-support tools may improve uptake and understanding among clinicians and patients.

## Conclusion

Current selection and positioning of IBD therapeutics are based on broad, clinical, biochemical, radiological and endoscopic profiling. Understanding the underlying molecular drivers of IBD may inform selection of more effective therapy in the pursuit of precision medicine (figure 2). Despite recent progress, the vast majority of existing biomarkers to predict IBD treatment response have not been incorporated into clinical practice. Future technological advances in both experimental techniques, machine learning and collaborative research will help to address these deficiencies. Once accurate biomarker predictors are identified, measuring biomarkers for treatment response must be affordable and widely available to ensure equitable access to precision medicine to improve the quality of life of patients with IBD.
